# Live-cell imaging of compartment-specific redox changes in menkes disease fibroblasts

**DOI:** 10.1186/1755-8166-7-S1-P54

**Published:** 2014-01-21

**Authors:** Ashima Bhattacharjee, Martina Ralle, Svetlana Lutsenko

**Affiliations:** 1 Johns Hopkins School of Medicine, Baltimore, Maryland, USA; 2 Oregon Health and Science University, Portland, USA

**Keywords:** Copper, Redox, Glutathione, ATP7A, Menkes

## Background

Copper is an essential micronutrient and its misbalance in the body is associated with severe neurodegenerative disorders. While the importance of copper in the body is evident, mechanisms by which copper misbalance induces pathologic changes and disease symptoms are poorly understood. In this study, using fibroblasts from Menkes disease patient, as a cellular model of copper accumulation, we examined whether excess copper triggers specific and distinct changes in the redox environment of different cellular compartments.

## Subjects and methods

Skin fibroblasts from Menkes disease patient (YS cells, ATP7A^-/-^) and his heterozygous mother (ATP7A^+/-^) were used as an experimental system. Glutathione mediated redox environment and levels of H_2_O_2_ were investigated in nuclei, cytosol, and mitochondria of live cells by tagging the respective ratiometric sensors (GRX-roGFP and HyPer) with a compartment-specific localization signal.

## Results

Under basal conditions, the YS and XS cells show similar glutathione mediated redox environment in the nucleus and cytosol. However, the mitochondria are oxidizing in YS cells. YS cells were observed to accumulate higher level of peroxide in the cytosol and mitochondria. We also found that copper accumulation in cytosol and nuclei of YS cells sensitize cells to glutathione depletion suggesting importance of glutathione in protection of cells against copper overload

## Conclusion

Our experiments revealed differential response of cellular compartments to excess copper in cells. Nuclei, in spite of being a site of copper accumulation, do not show marked redox changes, suggesting presence of robust protective mechanisms operating in this compartment. H_2_O_2_ accumulation in cytosol in YS cells does not change glutathione balance, whereas mitochondria appear most affected, since both H_2_O_2_ levels and glutathione balance are altered. We propose that mitochondria could be a primary site of copper toxicity in Menkes fibroblasts. Molecular mechanisms underlying differential redox responses are presently being investigated.

